# Brucellosis Seropositivity in Animals and Humans in Ethiopia: A Meta-analysis

**DOI:** 10.1371/journal.pntd.0005006

**Published:** 2016-10-28

**Authors:** Getachew Tadesse

**Affiliations:** Department of Biomedical Sciences, College of Veterinary Medicine and Agriculture, Addis Ababa University, Debrezeit, Ethiopia; Texas A&M University College Station, UNITED STATES

## Abstract

**Background:**

The objectives of this study were to assess the heterogeneities of estimates and to estimate the seroprevalence of brucellosis in animals and humans in Ethiopia.

**Methods/Principal findings:**

Data from 70 studies covering 75879 animals and 2223 humans were extracted. Rose Bengal Plate Test (RBPT) and Complement Fixation Test (CFT) in series were the most frequently used serological tests. A random effects model was used to calculate pooled prevalence estimates. The overall True Prevalence of brucellosis seropositivity in goats and sheep were estimated at 5.3% (95%CI = 3.5, 7.5) and 2.7% (95%CI = 1.8, 3.4), respectively, and 2.9% for each of camels and cattle. The prevalence was higher in post-pubertal than in pre-pubertal animals (OR = 3.1, 95% CI = 2.6, 3.7) and in the pastoral than in the mixed crop-livestock production system (OR = 2.8, 95%CI = 2.5, 3.2). The incidence rates of brucellosis in humans of pastoral and sedentary system origins were estimated at 160 and 28 per 100 000 person years, respectively.

**Conclusions:**

The seroprevalence of brucellosis is higher in goats than in other species. Its occurrence is evocative of its importance in the country in general and in the pastoral system in particular. Public awareness creation could reduce the transmission of *Brucella* spp. from animals to humans and the potential of livestock vaccination as a means of control of brucellosis needs to be assessed.

## Author Summary

Brucellosis is one of the neglected zoonotic diseases with major outcomes of reproductive wastage in livestock and debilitating illnesses in humans. Ethiopia is one of the sub-Saharan African countries with the largest animal and second largest human population. The aim of this study was to estimate the occurrence of brucellosis in animals and humans. To this effect, data from 70 published studies were extracted, and meta-analytical methods were employed. Of the domestic animals, the highest prevalence was in goats (5.3%), and the lowest in sheep (2.7%). Brucellosis seropositivity was higher in animals of the pastoral than the mixed crop-livestock production system. In humans, the prevalence was estimated at 17.4% in the pastoral and 3.1% in the sedentary system, and the incidence rates, respectively, were 160 and 28 per 100 000 person years. The high occurrence of the disease is evocative of its importance in the country in general and in the pastoral system in particular. Public awareness creation could help reduce its occurrence in humans, and the potential of livestock vaccination as a means of control needs to be assessed.

## Introduction

Brucellosis is one of the neglected zoonotic diseases, and there have been several reports that addressed its global importance [1–3]. The transmission of *Brucella* spp. of zoonotic importance—*Brucella melitensis*, *Brucella abortus*, *Brucella suis* and *Brucella canis—*is either horizontal or vertical in animals, and in humans it is mainly related to contact with infected animals or ingestion of unpasteurized milk or products thereof [4]. Eradicated or controlled in most developed nations, it remains important in poverty stricken countries, and reproductive wastage and debilitating illnesses in livestock and humans, respectively, are its major outcomes [1–3]. Moreover, problems associated with its therapeutic management [5], demographic shifts, and substantial increases in its complications [6], persistence in pastoral communities [7], and an increased prevalence in areas where small ruminants predominate [8] have been reported. However, despite reports on its significance, large scale national level prevalence and incidence estimates in sub-Saharan countries where it could have a substantial impact are sparse.

Ethiopia is one of the sub-Saharan African countries, with the largest animal and second largest human population. Livestock are the major sources of income and security for a significant segment of the population, and the system of animal production is by and large of an extensive type. The relationship between humans and animals is so close, and the perception of the general population as regards risky practices that favor pathogen transmission is low. Although brucellosis has been reported to be endemic [9], rigorous country wide statistics are lacking. The objectives of this study were to assess the heterogeneities of estimates, and estimate the pooled seroprevalence of brucellosis in animals, and humans by using meta-analytical methods.

## Methods

The PRISMA (Preferred Reporting Items for Systematic Reviews and Meta-Analyses) guideline was used in the analysis [10], and the checklist was used to ensure inclusion of relevant information (S1 Checklist). The outcomes of interest were seroprevalence estimates of brucellosis in animals and humans, and the questions addressed were the following: (i) number, diversity, and scope of studies carried out so far, (ii) heterogeneities of estimates, (iii) pooled prevalence estimates, and (iv) factors associated with the occurrence of brucellosis.

### Search and selection of studies

Medline in the Pub Med interface, Google Scholar, Google, African Journals online (AJOL) and lists of references of articles were used in the search for studies. The languages of publication were restricted to English and French. Brucell* AND Ethiopia in English, and Brucell* ET Éthiopie in French were the keywords used in electronic searches. To retrieve additional articles, the keywords were combined with other words that included humans, cattle, sheep, goats, camels, pigs, dogs, and horses. The last search was done on August 10, 2016. Eligible studies were selected by using inclusion and exclusion criteria. A study was eligible if it fulfilled the following criteria: (i) full text and published in English or French, (ii) carried out on animals and/or humans after 2000, (iii) cross-sectional/ frequency, (iv) employed enzyme immunoassay methods or Rose Bengal Plate Test (RBPT) and Complement Fixation Test (CFT) in series. Initially, studies with titles and/or abstracts that were not relevant to the outcomes of interest were excluded. In the succeeding steps, reviews, case reports, studies carried out before 2000 and unavailable were excluded, and full text reports were screened for eligibility. Exclusion of screened reports was done by the following criteria: duplication (articles/data), Rose Bengal Plate Test (RBPT) only (to reduce bias/ method related heterogeneity associated with lower specificity of RBPT), farm based report, sample size (< 100 in animal studies), and inconsistent data (data incoherent within a table or tables or in the narrative sections, and could not be figured out).

### Data extraction

The following data were extracted from eligible studies: first author, year of publication, year of study, location (region), altitude (lowland, midland and highland), species (camel, cattle, sheep, goat, and humans), production system (pastoral/agro-pastoral, mixed crop-livestock, intensive/semi-intensive), sample size (herd/animal), sampling methods (probability/non-probability based), breed (local, cross/exotic), age group (depending on the species), sex (male/female), serological test methods, the laboratory where the serological tests were done, and the number of samples examined and positive. To stabilize the variance, the study level estimates were transformed to double arcsine estimates (t) by the following formulae: t = sin^-1^(√n/N+1) + sin^-1^(√n+1/N+1), and se (t) = √1/N+0.5 [11]. Logit transformations were used to estimate odds ratios.

### Data analysis

The data was analyzed by using Stata (Version 11.1, Stata Corp, College Station, Texas), Epi Info^TM^ (Version 3.5.1, Center for Disease Control, CDC, USA) and Microsoft Office Excel 2007. Alpha was set at 0.05, and in multiple comparisons the Bonferroni correction method was applied. Data from animals and humans were analyzed separately.

### Bias assessment

In animal studies, the within study bias was assessed by quality item [12] and quality index measures [13]. The quality items used to measure external validity were sampling methods, sample size, and data reporting quality. A value of one was assigned for reporting each of the following, but none otherwise: probability based sampling method (herd/flock and animal), study period, herd number, laboratory where the serological tests were done, system of production, breed, age and sex as per the set objectives. The worth for sample size was calculated by dividing the sample size (n) by 384. The quality index was calculated by dividing the total score with the highest score as a denominator. Whether prevalence estimates were associated with studies’ qualities were assessed by quality item and quality index regression analyses. A funnel plot was used to visually examine the across study bias (small study effects). The Egger’s regression asymmetry test was used to test the significance of the plot’s asymmetry. Cumulative meta-analyses were performed to examine the magnitude of changes in pooled estimates.

### Heterogeneity assessment

Heterogeneity analyses were done to assess the variability among study level estimates. The Galbraith plot was used to graphically examine the heterogeneity of estimates. Inverse variance and Mantel-Haenszel models were used to estimate the heterogeneities of prevalence and odds ratios estimates, respectively. The Cochran’s Chi squared test (Q-test), tau squared (T^2^), and Higgin’s statistics/indexes (H^2^ and I^2^) [14] were used to measure heterogeneities. I^2^ estimates less than 25% and greater than 75%, respectively, were considered as low and high heterogeneities. The 95% confidence bounds of I^2^ were derived from the H statistics: ln (H) +/-1.965 (Se ln H). Logit estimates of ‘H’ were back transformed to I^2^ by the following formula: I^2^ = 1-1/H^2^. If Cochran’s Q was greater than the number of studies (k), the standard error (Se) of I^2^ was calculated by the following formula: Se (ln H) = 0.5 [(ln Q-ln k-1) / (√2Q-√2k-3)]. If the number of studies was greater than Cochran’s Q, the standard error of I^2^ was calculated by the following formula: Se (ln H) = √ [(1/2k-4) (3(k *−* 2)^2^–1) / (3(k *−* 2)^2^)], [14].

### Subgroup analyses

Subgroup analyses were done on the basis of assumed relative homogeneity due to biological or environmental factors, heterogeneity statistics, and data availability. Because of the small number of animal studies that employed enzyme immunoassays, and differences in tests’ performances, all subsequent analyses were performed by using data from studies that employed Rose Bengal Plate Test (RBPT) and Complement Fixation Test (CFT) in series. The categories and subgroups considered in animal studies were the following: species (camels, cattle, goats and sheep), puberty (post-pubertal and pre-pubertal), sex (male and female), and production system (pastoral, mixed crop-livestock and semi intensive/intensive). Additional subgroup analyses were done on the following: pastoral system by location (Afar, Borana, Somali and South Omo), camel by location (Afar, Borana and Somali), cattle by breed (local and cross) and production system (pastoral, mixed crop-livestock and semi-intensive/intensive), small ruminants by production system (pastoral and mixed crop-livestock) and by pastoral areas (Afar, Borana, Somali and South Omo). Data from South Eastern Tigray, North Eastern Oromia, Bale and Dire Dawa were grouped in the main pastoral locations depending on proximity (Afar, Borana and Somali). The age effect was assessed in the context of age at puberty. Due to disparities in the categorization of age groups and the difficulty to disaggregate aggregate data, cutoff values of four years for camels, three years for cattle, and one year (10 months to 18 months) for small ruminants were used to group animals as post-pubertal and pre-pubertal. Subgroup analyses by herd/flock size were not done because of lack of uniformity in herd/flock definition, and in numerical categorizations of size (small, medium and large), and due to the difficulties to disaggregate aggregate data as is the case in age groups. Subgroup analyses by agro climatic zones (lowland, midland and highland) were not done due to lack of distinct data on the exact number of animals sampled and positive by climatic zone in most cases in point, and on account of the potential climatic heterogeneity in areas where a mixed crop-livestock production is practiced. As the number of eligible human studies was small and the residual heterogeneity of the tests (CFT vs. other tests) was not significant (P > 0.05), the overall pooled estimate was calculated by using all data irrespective of serological tests. Subgroup analyses were performed by production system (pastoral and mixed crop-livestock/semi-intensive/intensive) and occupation (slaughterhouse personnel/butchers/animal health and production workers).

### Prevalence estimation

Forest plots were used to graphically display estimates. The DerSimonian and Laird random effects model was used to pool double arcsine estimates. The sensitivities of pooled estimates were assessed by single study omitted influence analyses. Pooled double arcsine estimates were back transformed to prevalence estimates (p) by the following formula: p = 0.5 {1-sgn (cos t) [1- (N (sin t)^2^ + (sin t)^2^–1/ (N sin t))^2^]^0.5^}, [11], where N = sample size. Log odds estimates were back transformed to odds ratios.

### Stratified and meta-regression analyses

The significance of the residual heterogeneity within subgroups was assessed by the Chi distribution (Overall Q - ∑Q of subgroups, df). The Yates’ Chi square was used to test the significance of a difference between pooled estimates. A meta-regression was performed to search for variables that best explain the heterogeneity and predict brucellosis seropositivity in animals. For this purpose, 21 records with data on age at puberty and sex were considered. Due to the limited number of studies considered for the regression analysis, only age at puberty and sex were considered as covariates. Initially, whether the sampling proportions (post pubertal, and female) were associated with prevalence estimates were examined. The dependent variable in the regression proper was a double arcsine prevalence estimate, and the covariates were raw prevalence differences in post-pubertal and pre-pubertal animals, in females and males, and an age-sex interaction term. Tau squared (T^2^) was estimated by the Residual Maximum Likelihood (REML) method and the probability was calculated by the Monte-Carlo permutation test at 20000 iterations.

### True prevalence and incidence estimation

To estimate True Prevalence in cattle and small ruminants, the sensitivity and specificity estimates of each of RBPT and CFT predicted by a Bayesian logistic regression model [15] were imputed in the Rogan and Gladen formula [16]: Tp = (Ap + CSp_s_-1)/(CSe_s_+CSp_s_-1), where Tp = true prevalence; Ap = apparent prevalence; CSe_s_ = combined sensitivity of the test series (Se RBPT × Se CFT), and CSp_s_ = combined specificity of the test series (1-(1-Sp RBPT) × (1-Sp CFT)). The sensitivity and specificity estimates of RBPT and CFT in cattle and small ruminants were the following [15]: RBPT in cattle (Se = 0.981(95%CI = 0.968,0.991) and Sp = 0.998 (95%CI = 0.997,0.998)); RBPT in small ruminants (Se = 0.925 (95%CI = 0.916,0.934) and Sp = 0.999(95%CI = 0.998,1.000)); CFT in cattle (Se = 0.96 (95%CI = 0.949,0.970) and Sp = 0.998 (95%CI = 0.997,0.998)); CFT in small ruminants (Se = 0.926 (95%CI = 0.911,0.939) and Sp = 0.999 (95%CI = 0.998,0.999)). Estimates of the sensitivities and specificities of each of RBPT and CFT in camels, and humans are divergent and pooled estimates are not available. Both RBPT and CFT are applicable in camels and humans; the sensitivity of RBT in humans is over 99%, and CFT reportedly has a positivity of 91.7% [17]. Due to the discrepancy in study level estimates and in the absence of pooled estimates, the performances of the tests were assumed to be as equally good as in cattle and small ruminant sera. Therefore, to estimate True Prevalence in camels, humans, and in subgroups with mixed animal species, the sensitivity and specificity estimates of each of RBPT and CFT [15] were separately pooled by a random effects model -using the median estimates and 95% confidence limits [15] as inputs. The calculated pooled estimates (Se RBPT = 0.953; Sp RPBT = 0.998; Se CFT = 0.943; Sp CFT = 0.999) were imputed in the Rogan and Gladen formula. The incidence of brucellosis in humans was calculated by using the relationship between prevalence (p) and seroconversion (a), and loss of seropositivity at equilibrium (b): p = a / (a + b), [18,19], where p = calculated True Prevalence, a = 0.1, and b = 0.092 [18].

## Results and discussion

### Search and selection of studies

**Fig 1:**
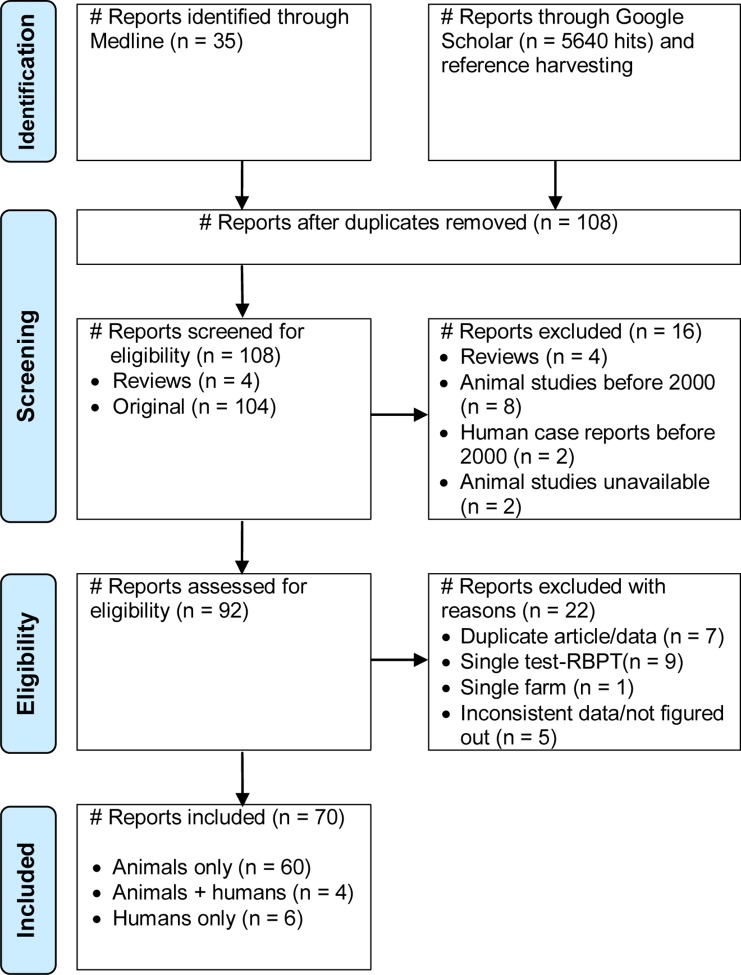
Flow diagram of search and selection of studies.

Fig 1 presents a flow diagram of the search and selection of studies. Of 108 reports identified, 104 were original and 4 were reviews (3 narratives, and one systematic on dairy cattle). Two articles were in French and the rest were in English. Reports published in languages other than English and French have not been noted. Nine original reports were on non ruminants (7 on humans only, one on pigs and one on horses- *Brucella abortus* titer and fistulous withers); 90 were on domestic ruminants only, and five on ruminants and humans. At the screening stage, 16 reports were excluded: reviews (4), studies carried out before 2000 (10), and unavailable (2). Out of 92 full text reports assessed for eligibility, 22 (23.9%) were excluded for diverse reasons: duplication-article/data (7), single test (9), single farm (1), and inconsistent data (5), (S2 Table). Altogether, data from 70 studies with 98 species wise records (15 camels, 27 cattle, 21 sheep, 25 goats and 10 humans) were extracted [20–89].

The number, diversity and scope of studies carried out so far are inadequate. Although there has been a substantial increase in the number of studies since 2000, studies that addressed brucellosis in cattle of the pastoral system and in small ruminants of Somali and South Omo pastoral areas are relatively sparse. Data on livestock of Gambela (Western Ethiopia) is not available. Only one study has reported the prevalence of porcine brucellosis [90]. The roles of equines, wildlife and domestic dogs in the epidemiology of brucellosis have not been described. With the exception of one study that reported *B*. *melitensis* from slaughtered goats (2/14) [75], information on the species and biovars of *Brucella* is not available. The impact of brucellosis on ruminant productivity, and the potential costs and benefits of livestock vaccination have not been reported. Estimates of the incidence and impact of the disease in humans are non-existent, and the seroprevalence studies are generally limited in number and constrained by sample sizes.

### Characteristics of included studies

The characteristics of the studies are given in S1 Table. The studies have been carried out from 2000 to 2015 in Central, Western, Eastern, Northern and Southern Ethiopia. The species of animals were camels, cattle, goats and sheep, and humans. All animals and humans were unvaccinated against *Brucella* spp. and above six months of age, but in one study on goats (>4 months) [63]. The systems of animal production included pastoral, mixed crop-livestock and semi-intensive/intensive. The sample sizes ranged from 291 to 6201 in animals, and from 38 to 653 in humans. In total, data from 75879 animals and 2223 humans were considered for quantitative syntheses. RBPT-CFT in series were the most frequently used serological tests in animal (58/64) and human studies (7/10). Enzyme immunoassays were used in six animal studies. Immunochromatographic lateral flow assay, 2-mercaptoethanol, and slide agglutination test (Huma Tex febrile antigens test kit) have been used in three human studies. In most animal (53/64) and human (7/10) studies, the complement fixation tests have been done at the National Veterinary Institute and/or the National Animal Health and Disease Investigation Center, Ethiopia.

### Within and across study bias

**Fig 2:**
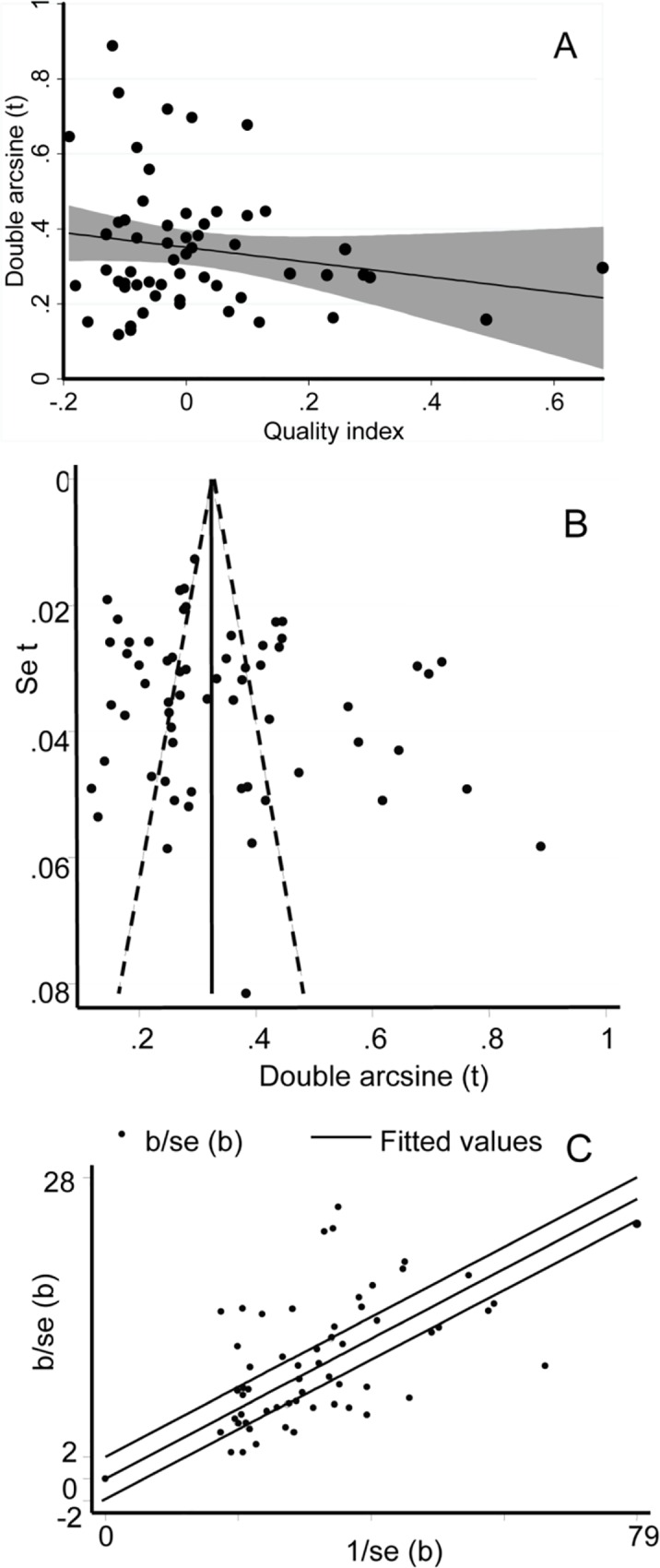
Plots of bias and heterogeneity in animal studies. (A) Regression plot of prevalence and study quality index. (B) Funnel plot. (C) Galbraith plot.

The quality items’ scores and the quality indexes of animal studies were not associated with prevalence estimates (P > 0.05), (Fig 2A). The funnel plot (Fig 2B), the Egger’s regression asymmetry test (b = 3.19, 95%CI = -0.28, 6.67; p > 0.05), and cumulative analyses did not suggest bias. Sample sizes have been calculated by considering expected prevalence and desired precision. In most instances the calculated sample sizes were inflated to account for potential intra-cluster variability (S1 Table). The overall sampling proportions tended to be higher in post-pubertal than in pre-pubertal and in female than in male animals. The operational definitions and classifications of herd/flock size and age groups lack uniformity across studies. Non-reporting of exact sampling methods, incomplete and/or aggregate data reporting were among the shortcomings. The farming practice in the extensive system apparently set hurdles to perform a random sampling in the strictest sense of a probability based sampling. However, as the sampled animals were drawn from different owners or herds/flocks and without *a priori* knowledge on the seropositivity/ seronegativity status of the units, the bias likely to have been introduced due to ‘non-probability sampling’ could be considered unimportant. Furthermore, most CFT have been done in two certified laboratories, and the bias and heterogeneity due to CFT could be hold negligible. Nonetheless, differences associated with the sampling proportions by age group and sex could have contributed to the between studies’ variability.

### Heterogeneity estimates

Most estimates on animal brucellosis lie beyond the confidence limits of the regression line of the Galbraith plot (Fig 2C). The study level estimates were heterogeneous (P < 0.0001), and in a subgroup analysis by serological tests, the residual heterogeneity was significant (P < 0.0001). The subgroup heterogeneity estimates are presented in Tables 1 and 2. Species wise, the highest between-study variance (T^2^) was in goats. By system, T^2^ was higher in cattle of the semi-intensive/intensive system than in the mixed crop-livestock and in small ruminants of the pastoral system than in the mixed crop-livestock. The I^2^ and/or the lowest 95% uncertainty limits were low to moderate-high in most subgroups with less than eight studies (7/11). In all other animal subgroups but animal-pre-pubertal and cattle-mixed crop-livestock subgroups, the I^2^ estimates including the lower confidence bounds were substantially high (I^2^ > 75%).

**Table 1:** Heterogeneity estimates in animals and humans.

Category		Subgroup	Nr	Ns	Q	Q-P	T^2^^†^	H^2^	I^2^,95% CI		
Animals	Species	Camel	14	14970	163	0.0001	0.011	12.5	0.92	0.88	0.95
		Cattle	23	25579	464	0.0001	0.018	21.1	0.95	0.94	0.96
		Goat	21	15957	508	0.0001	0.035	25.4	0.96	0.95	0.97
		Sheep	19	13261	202	0.0001	0.016	11.2	0.91	0.88	0.94
	Puberty	Post P	39	33985	626	0.0001	0.018	16.5	0.94	0.93	0.95
		Pre P	26	9775	93	0.0001	0.007	3.7	0.73	0.60	0.82
	Sex	Female	49	43475	791	0.0001	0.018	16.5	0.94	0.93	0.95
		Male	39	22459	241	0.0001	0.01	6.3	0.84	0.79	0.88
	Breed C	Cross	11	8561	178	0.0001	0.023	17.8	0.94	0.91	0.95
		Local	15	14627	216	0.0001	0.015	15.3	0.94	0.92	0.96
	System	Mixed-CL	19	17328	120	0.0001	0.006	6.7	0.85	0.78	0.90
		Pastoral	30	36239	851	0.0001	0.024	29.3	0.97	0.96	0.97
		Si/I	12	9748	189	0.0001	0.021	17.2	0.94	0.92	0.96
Humans	System	Pastoral	3	396	14	0.001	0.048	6.9	0.86	0.57	0.95
		Sedentary	4	570	5	0.147	0.006	1.8	0.44	0	0.81
	Occupation	SBHP	4	445	10	0.023	0.026	3.2	0.69	0.09	0.89
		Overall	10	2223	104	0.0001	0.051	11.6	0.91	0.86	0.95

Breed C, cattle breed; H^2^ & I^2^, Higgin’s statistics/ indexes; Mixed-CL, mixed crop-livestock production system; Nr, number of reports; Ns, number of samples; Post P, post-pubertal; Pre P, pre-pubertal; Q, Cochran’s Chi squared; Q-P, Cochran’s P value; Si/I, semi intensive and intensive production system; SBHP, slaughterhouse personnel, butchers, animal health and production workers; T^2^, tau squared.

^†^Double arcsine estimate.

**Table 2:** Heterogeneity estimates by species and system or location.

Category	Subgroup	Nr	Ns	Q	Q-P	T^2^^†^	H^2^	I^2^,95% CI		
Pastoral AA	Afar	15	13130	264	0.0001	0.021	18.9	0.95	0.93	0.96
	Borana	11	14852	109	0.0001	0.008	10.9	0.91	0.86	0.94
	Somali	11	6792	134	0.0001	0.021	13.4	0.93	0.89	0.95
	South Omo	3	1183	20	0.0001	0.025	9.7	0.90	0.72	0.96
Camel	Afar	7	4301	17	0.01	0.003	2.8	0.64	0.19	0.84
	Borana	6	7783	204	0.001	0.003	4	0.75	0.43	0.89
	Somali	5	2886	3	0.493	0	0.9	0.00	0	0.76
Cattle	Pastoral	4	2045	31	0.0001	0.019	10.4	0.90	0.78	0.96
	Mixed-CL	11	9637	65	0.0001	0.006	6.5	0.85	0.74	0.91
	Si/I	12	9748	189	0.0001	0.021	17.2	0.94	0.92	0.96
Sheep/goats	Pastoral	16	19224	641	0.0001	0.038	42.7	0.98	0.97	0.98
	Mixed-CL	8	7691	48	0.0001	0.006	6.8	0.85	0.73	0.92
	Afar	9	8829	230	0.0001	0.031	28.8	0.97	0.95	0.98
	Borana	6	6223	47	0.0001	0.009	9.4	0.89	0.79	0.94
	Somali	4	3169	104	0.0001	0.054	34.6	0.97	0.95	0.98
	South Omo	2	1003	17	0.0001	0.034	16.9	0.94	0.81	0.98

H^2^, I^2^ = Higgin’s statistics/ indexes; Mixed-CL, mixed crop-livestock production system

Nr, number of reports; Ns, number of samples; Pastoral AA, all pastoral animals

Q, Cochran’s Chi squared; Q-P, Cochran’s P value; Si/I, semi intensive and intensive production system

T^2^, tau squared.

^†^Double arcsine estimate.

In subgroups with less than eight studies, the 95% uncertainty intervals of I^*2*^ demonstrate high degree of uncertainties, and the low to moderate estimates do not warrant the absence of substantially high heterogeneities. Furthermore, in all subgroups with less than eight studies but human-sedentary and camel-Somali, the Q test shows the presence of a significant heterogeneity even at a 10% cutoff level. The high heterogeneities could be attributed to multiple factors that may involve biological (*Brucella* spp., host species, breed, age and sex), environmental (agro-ecology, livestock composition and management practice) and interactions of factors. Differences in the sampling proportions of age groups and female/male could also have contributed to the heterogeneities. Due to the small number of studies, and incomplete and/or aggregate data in several studies, strata based classifications (sub -sub grouping) and further assessments could not have been performed. However, the comparatively higher differences between the largest and smallest T^2^ estimates in species and production system subgroups than in other subgroups (age, sex and breed) imply the importance of species and production system as major moderators of heterogeneity. Moreover, as quantification is only one component in variability assessment, and the most important aspect being the implication of the heterogeneity [91], the species and production system could be considered as epidemiologically most important sources of variability.

Each of the measures of heterogeneity has its merits and demerits. The Q-test has a low power to detect heterogeneity in small number of studies but an excessive power when the number of studies is large [14, 91, 92]. As sample size (study precision) increases, both Q and I^2^ increase [92] and suggest the presence of unimportant heterogeneity. Furthermore, due to the dependence of I^2^ on the number and precisions of studies, the 95% uncertainty intervals may not retain the I^2^ coverage [93], and the cut off values- low, moderate, and high- are generally arbitrarily defined [14,91]. Although, T^2^ is inherently unaffected by the number and precisions of studies [92], its interpretation is difficult, and does not facilitate comparison across different outcome measures [14]. On the whole, the median number (inter quartile range) of studies included in Cochrane and non Cochrane meta-analyses, respectively, have been reported to be 16 (7–30), and 8 (4–16) [94]. However, what exactly are ‘small’ / ‘large’ numbers of studies and sample sizes in meta-analyses have not been adequately described [93].

### Prevalence estimates

**Fig 3:**
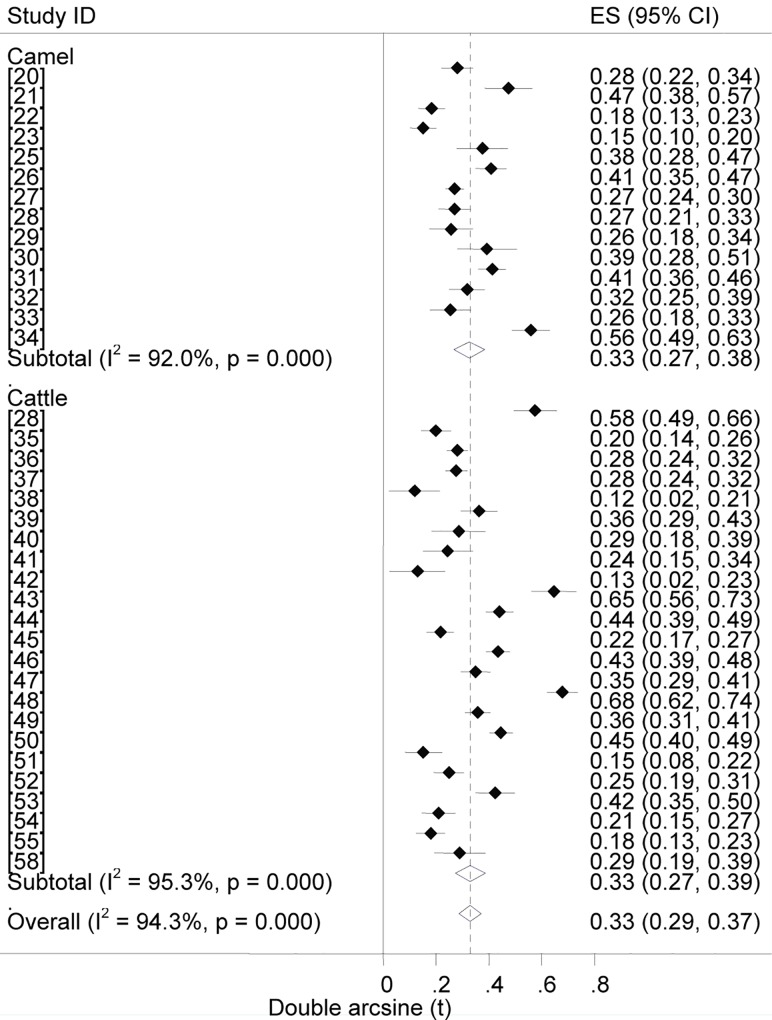
Forest plots of prevalence of brucellosis seropositivity in large ruminants.

**Fig 4:**
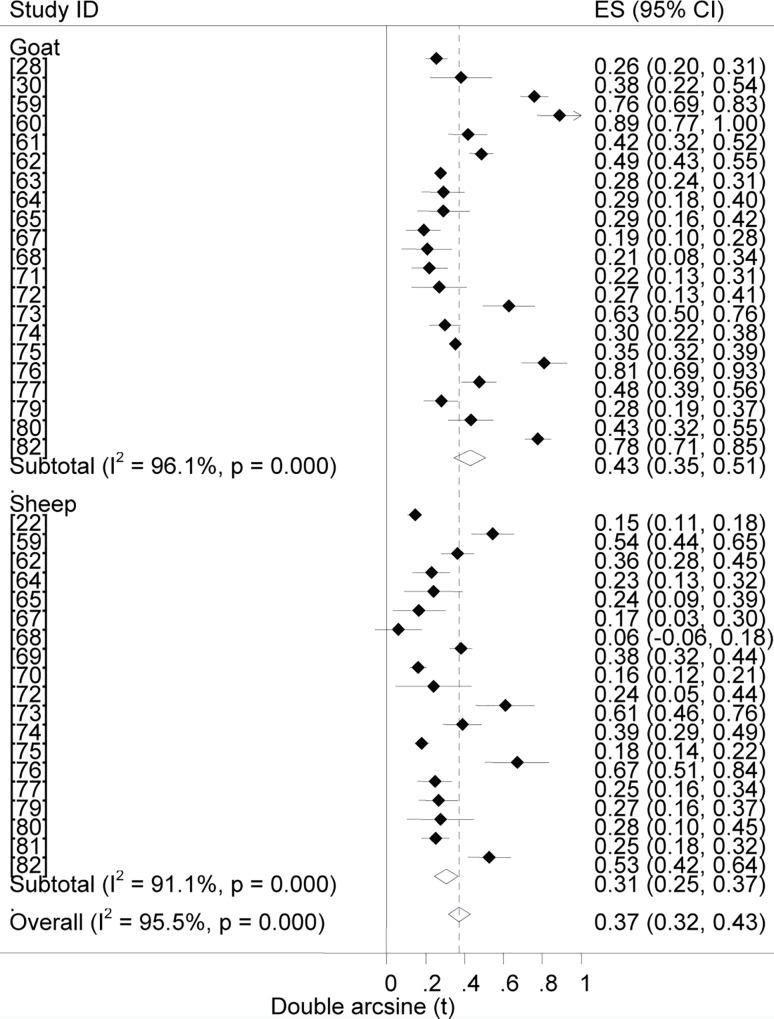
Forest plots of prevalence of brucellosis seropositivity in small ruminants.

Forest plots of the prevalence of brucellosis seropositivity in large and small ruminants are presented in Figs 3 and 4, respectively. The subgroup prevalence estimates are presented in Tables 3 and 4. The overall pooled prevalence of animal brucellosis was estimated at 3% (95% CI = 2.4, 3.6). All single study omitted pooled estimates lie within the 95% confidence bounds of the respective overall means. In most subgroups the 95% confidence intervals of the pooled estimates imply low uncertainties in the location of the respective means.

**Table 3:** Pooled prevalence of brucellosis seropositivity in animals and humans.

Category		Subgroup	Nr	Ns	Prevalence, 95%CI			P	TP, 95% CI		
Animals	Species	Camel	14	14970	2.6	1.8	3.7	0.0001	2.9	2.0	4.1
		Cattle	23	25579	2.7	1.8	3.7	0.0001	2.9	1.9	3.9
		Goat	21	15957	4.5	3.0	6.4	0.0001	5.3	3.5	7.5
		Sheep	19	13261	2.3	1.5	3.4	0.0001	2.7	1.8	3.4
	Puberty	Post P	39	33985	3.8	2.9	4.7	0.0001	4.2	3.2	5.2
		Pre P	26	9775	1.3	0.8	1.7	0.0001	1.4	0.9	1.9
	Sex	Female	49	43475	3.4	2.7	4.1	0.0001	3.8	3.0	4.6
		Male	39	22459	1.9	1.4	2.4	0.0001	2.1	1.6	2.7
	Breed C	Cross	11	8561	3.1	1.6	4.9	0.0001	3.3	1.7	5.2
		Local	15	14627	2.6	1.6	3.7	0.0001	2.8	1.7	3.9
	System	Mixed	19	17328	1.5	1.1	2.1	0.0001	1.7	1.2	2.3
		Pastoral	30	36239	4.2	3.1	5.4	0.0001	4.7	3.4	6
		Si/I	12	9748	3.1	1.8	4.7	0.0001	3.4	2.0	5.2
Humans^†^	System	Pastoral	3	396	21.1	11.3	33	0.0001	17.4	12.9	22.4
		Sedentary	4	570	3.6	1.7	6	0.0001	3.1.	0.9	6.5
	Occupation	SBHP	4	445	2.1	0.2	5.9	0.003	1.2	0	5.7
		Overall	10	2223	6.4	3.3	11	0.0001	6.7	2.4	12.8

Breed C, cattle breed; Mixed-CL, mixed crop livestock production system; Nr, number of reports; Ns, number of samples; Post P, post pubertal; Pre P, pre pubertal; Si/I, semi intensive and intensive production system; SBHP, slaughterhouse personnel, butchers, animal health and production workers; TP, True prevalence.

^†^The True Prevalence estimates were calculated based on RBT-CFT data.

**Table 4:** Pooled prevalence of brucellosis seropositivity by species and system or location.

Category	Subgroup	Nr	Ns	Prevalence, 95%CI			P	TP, 95% CI		
Pastoral AA	Afar	15	13130	6.8	5	8.9	0.0001	7.6	5.6	9.9
	Borana	11	14852	1.9	1.2	2.8	0.0001	2.1	1.3	3.1
	Somali	11	6762	2.4	1.2	4	0.0001	2.7	1.3	4.5
	South Omo	3	1183	2.4	0.3	6.1	0.001	2.7	0.3	6.8
Camel	Afar	7	4301	4.8	3.7	5.9	0.0001	5.3	4.1	6.6
	Borana	6	7783	1.2	0.7	1.8	0.0001	1.3	0.8	2
	Somali	5	2886	2	1.5	2.6	0.0001	2.2	1.7	2.9
Cattle	Pastoral	4	2045	5	2.4	8.5	0.0001	5.3	2.5	9
	Mixed	11	9637	1.7	1.1	2.5	0.0001	1.8	1.2	2.7
	Si/I	12	9748	3.1	1.8	4.7	0.0001	3.3	1.9	5.0
Sheep/goats	Pastoral	16	19224	5.2	3.2	7.5	0.0001	6.1	3.7	8.8
	Mixed-CL	8	7691	1.3	0.7	2.1	0.0001	1.5	0.8	2.1
	Afar	9	8829	8.4	5.4	12	0.0001	9.8	6.3	14
	Borana	6	6223	1.9	0.9	3.2	0.0001	2.2	1.1	3.7
	Somali	4	3169	2.6	0.2	7.5	0.007	3	0.2	8.8
	South Omo	2	1003	1.9	0.02	7.1	0.035	2.2	0.0	8.3

Mixed-CL, mixed crop-livestock production system; Pastoral AA, all pastoral animals

Nr, number of reports; Ns, number of samples; Si/I, semi intensive and intensive production system

TP, True Prevalence.

### Prevalence by animal species

The odds of brucellosis seropositivity was twice higher in goats than in sheep (OR = 2, 95%CI = 1.8, 2.3). The True Prevalence estimates for cattle and camels did not differ (2.9% each). The pooled estimates show the comparative importance of each of the species as reservoirs of *Brucella* spp. Although reports on the relative seroprevalence of brucellosis in species kept under a composite ownership are sparse, higher odds ratios of seropositivity in goats [63], and in camels and cattle [95] kept with one or more other species have been reported. The higher seroprevalence of caprine brucellosis suggests a higher potential of goats as source of *Brucella* spp. to other species with which they are kept, and this could be more evident in areas with high goat populations. Moreover, despite *B*. *melitensis* and *B*. *abortus*, respectively, being the classic causes of small ruminant and bovine brucellosis, their host specificity is relative [96], and elsewhere, there have been increasing evidence of *B*. *melitensis* infection in cattle [97], and small ruminant brucellosis due to *B*. *abortus* [96, 98–100]. Therefore, the general tendency of rearing mixed livestock species, and use of communal grazing resources in both the pastoral and mixed crop-livestock systems are indicative of the significance of both *B*. *melitensis* and *B*. *abortus* irrespective of the host species. As swine farming is a rare practice limited to urban and peri-urban set-ups, the role of *B*. *Suis* in ruminant brucellosis could be ruled out.

### Prevalence by puberty, sex and breed

**Fig 5:**
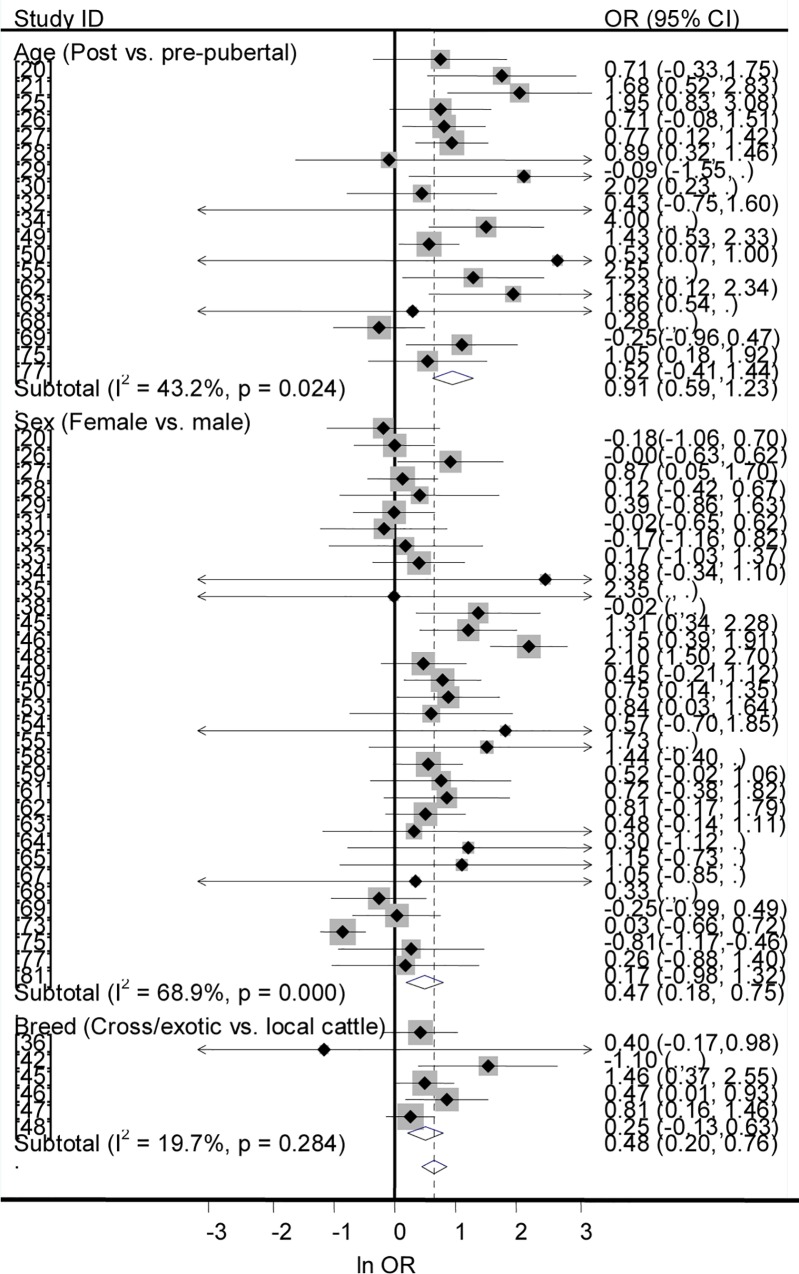
Forest plots of odds ratios of seroprevalence of brucellosis by puberty, sex and breed.

Forest plots of the odds of brucellosis seropositivity in post-pubertal vs. pre-pubertal animals, in females vs. males, and in improved vs. local cattle are presented in Fig 5. In the univariable analysis, the odds ratios of brucellosis seropositivity were higher in post-pubertal than in pre-pubertal animals (OR = 3.1, 95% CI = 2.6, 3.7), in females than in males (OR = 1.8, 95% CI = 1.7, 2), and in improved than in local cattle (OR = 1.2, 95% CI = 1.0, 1.4). In the multivariable analysis-the sampling proportions of each of post-pubertal and female animals were not associated with seroprevalence (P > 0.05) -puberty was significantly associated with brucellosis seropositivity (P < 0.001), (T ^2^ = 0.005, I^2^ = 84.6%, and R^2^ = 56.94%), but not sex (P > 0.05). Age at puberty varies by species, breed, sex and environmental factors. Although puberty is influenced by feed intake, reports on the impact of feed availability under different management scenarios are sparse, and the cutoff values are approximations of the performances reported for camels [101], cattle [102] goats [103] and sheep [104]. Due to the limited number of studies that met the inclusion criteria for a regression analysis, the model does not adequately explain the heterogeneity variance. Nonetheless, the calculated probability value is suggestive that post-pubertal animals are more likely to be seropositive than pre-pubertal animals. Despite the likelihood of exposure to *Brucella* spp. and brucellosis seropositivity apparently increase as age advances, the age effect could be modified by sex. The tendency of females to be slightly more seropositive than males could have been due to sampling bias rather than the effect of sex *per se*. As females are kept longer than males, the population of females is generally higher than that of males and more females have been sampled (Table 3). However, under natural breeding conditions males often search for females in heat -thus more exposed to infected females within or outside the herd/flock -and being a male may be a risk compared to being a female.

### Prevalence by production system

**Fig 6:**
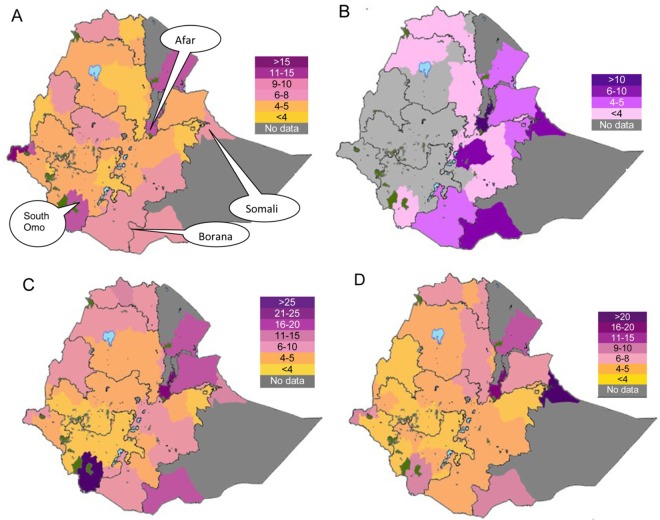
Average numbers of ruminants per holder. (A) Cattle. (B) Camels. (C) Goats. (D) Sheep.

Afar, Borana, Somali and South Omo are areas characterized by a non significant crop growing period [105], and the system of animal production is of a pastoral type (Fig 6A). The mixed crop-livestock production where it exists is practiced in areas typified by single or double crop growing periods. The semi-intensive/intensive system of production (dairy farming) is principally found in peri-urban and urban areas of the sedentary system. The populations and average numbers of cattle, camels, goats and sheep per holder vary within and among production systems (Fig 6), [106].

**Fig 7:**
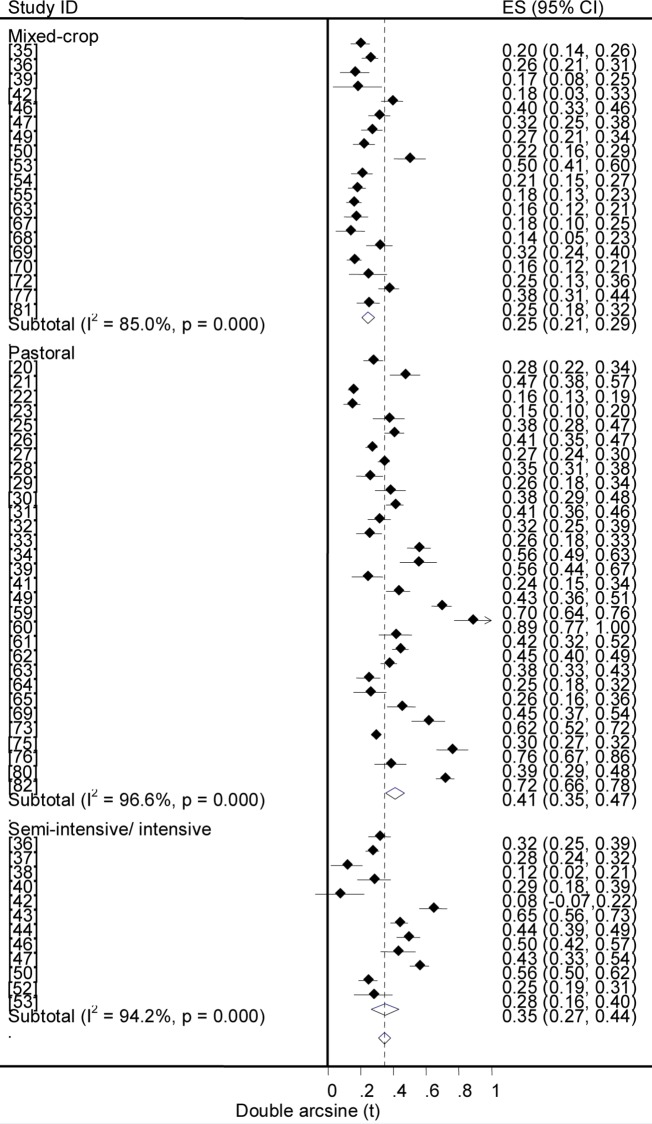
Forest plots of prevalence of brucellosis seropositivity in livestock by system.

**Fig 8:**
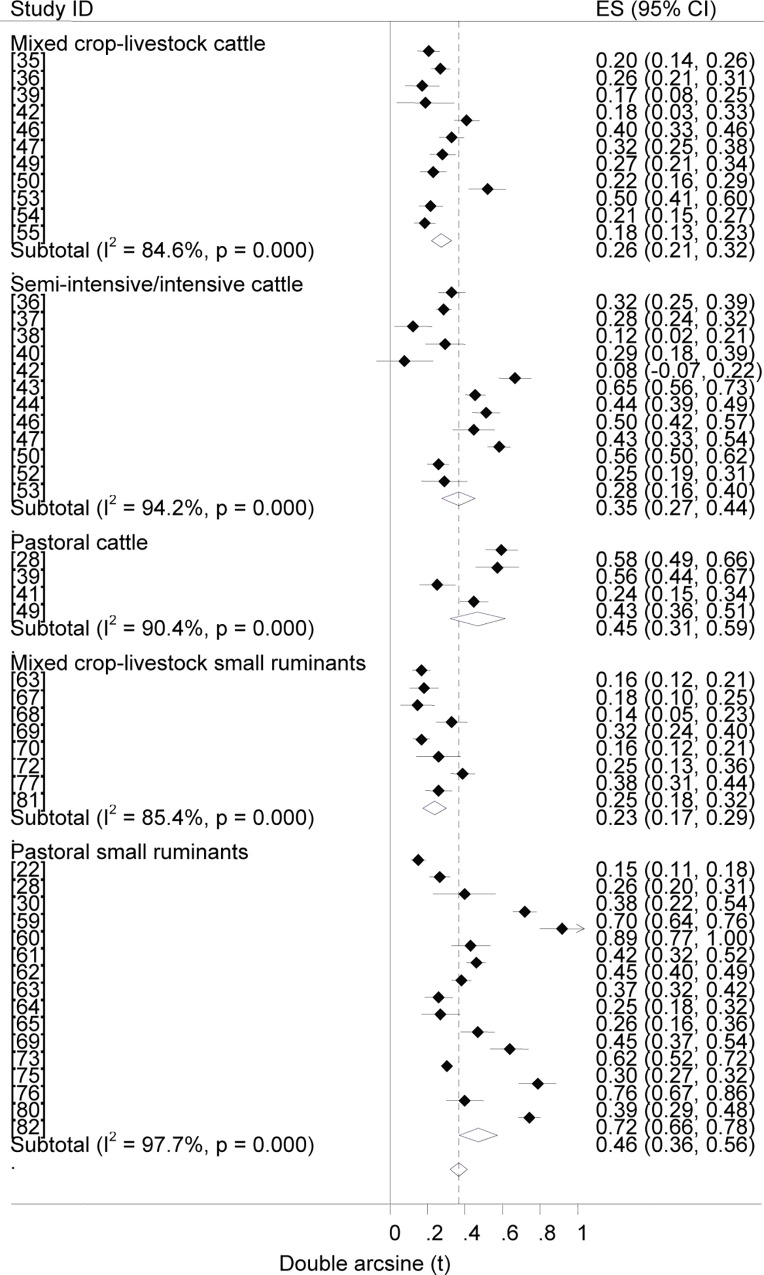
Forest plots of prevalence of brucellosis seropositivity in cattle and small ruminants by system.

Forest plots of the overall prevalence of brucellosis seropositivity in livestock by system are presented in Fig 7. The pooled estimates are given in Table 3. The prevalence of brucellosis seropositivity was higher in animals of pastoral origin than in animals from the mixed crop-livestock system (OR = 2.8; 95% CI = 2.5, 3.2), in small ruminants of the pastoral system than those from the mixed crop-livestock production system (OR = 4.3, 95% CI = 3.5,5.2), and in cattle under the semi intensive /intensive management system than in cattle from the mixed crop-livestock system (OR = 1.9; 95% CI = 1.5, 2.2), (Fig 8). Of the three systems, the highest prevalence was in pastoral animals. The comparable estimates of brucellosis seropositivity in cattle and small ruminants of the mixed crop-livestock system (Table 4) are suggestive of similar *Brucella* spp. transmission patterns. These similarities could form a basis on which to build study designs, irrespective of the heterogeneous micro climates within the sedentary system. The production system could influence the occurrence of the disease, and a higher risk in highly mobile and large herds than in less mobile and small herds has been reported [16].

### Prevalence by pastoral location

**Fig 9:**
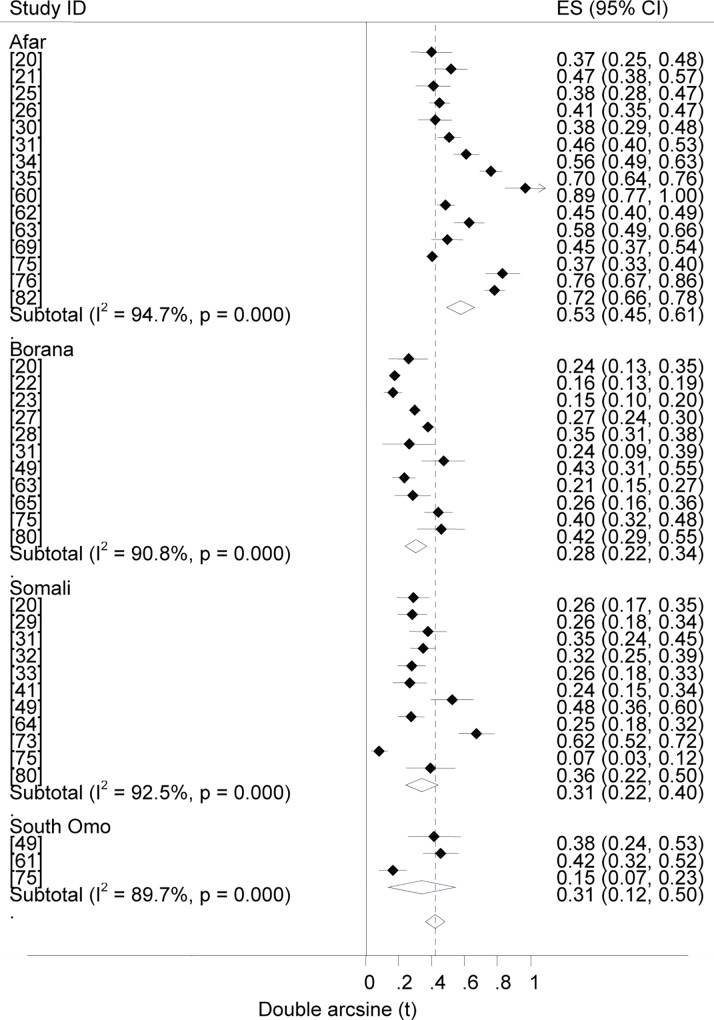
Forest plots of overall prevalence of brucellosis seropositivity in pastoral livestock by location.

**Fig 10:**
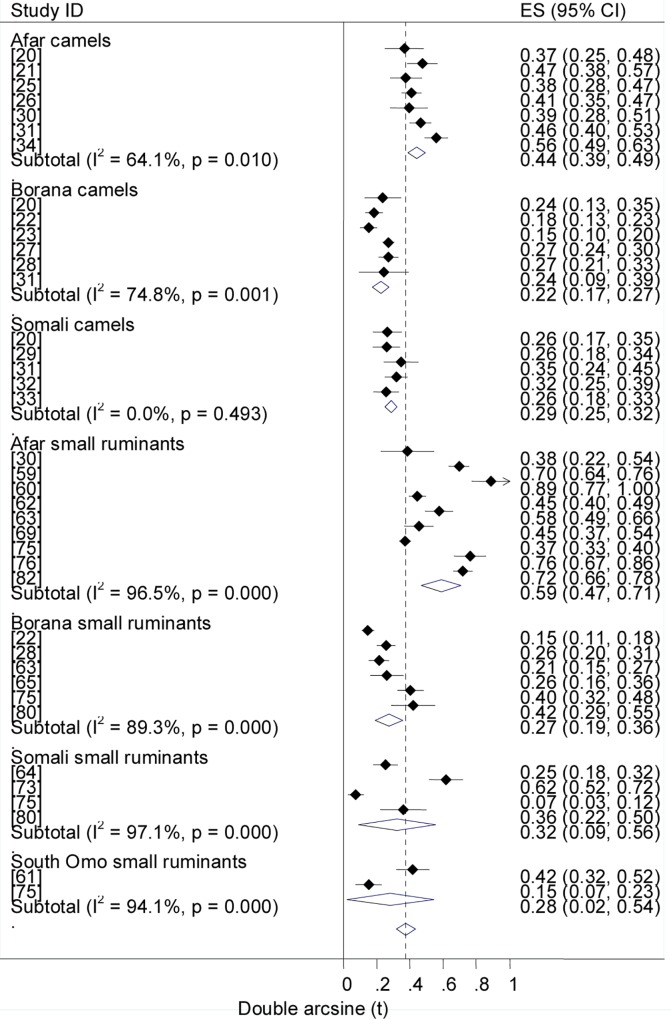
Forest plots of prevalence of brucellosis seropositivity in camels and small ruminants by pastoral location.

Forest plots of the overall estimates of brucellosis seropositivity in pastoral livestock by location are presented in Fig 9. The pooled estimates are given in Table 4. The odds ratios of brucellosis seropositivity were higher in Afar than in Borana livestock (OR = 3.8; 95% CI = 3.4, 4.4), in Afar than in Borana camels (OR = 4.3, 95% CI = 3.4, 5.4), and in Afar than in Borana small ruminants (OR = 4.8, 95% CI = 4, 5.7), (Fig 10). Compared to other pastoral locations (Borana, Somali and South Omo), the higher prevalence in Afar livestock suggests its importance in the region. The similarities of the estimates in pastoral areas other than Afar imply alike risk factors that may include the range and frequency of animal mobility, livestock species composition and herd/flock size. For instance, in Afar cattle and goats are preferred to sheep and camels, and animals from different localities could intermingle at grazing and watering points, but in Somali sheep and camels are the preferred species and the herding practice and use of resources is clan based [78]. Genetic makeup of animals may also account for the differences in estimates across pastoral locations. It has been reported that, whilst Maltese and South American sheep breeds demonstrated considerable resistance, Southwest Asian and Mediterranean fat-tailed sheep breeds were susceptible to brucellosis [107,108]. Prevalence estimates across pastoral areas could also differ depending on the stretch of the grazing area and the occurrence of brucellosis in wild animals. Although the significance of wildlife in the epidemiology of brucellosis has not been adequately described, *Brucella* spp. have been isolated from a variety of wild life [109], and an increased prevalence with increased density has been reported [110].

### Potential impact by production system

The impact of animal brucellosis is principally a function of prevalence/incidence, population size and effect on the productive /reproductive potential of the host, and its transmission to humans. The seroprevalence estimates (Tables 3 and 4) and the number of animals per holder (Fig 6A–6D) are higher in the pastoral than in the mixed crop-livestock system. While the overall proportion of improved cattle (0.74%) is lower than that of the indigenous [111], the herd size is higher in commercial and breeding farms [37] than in the mixed system. Despite reproductive wastage being a feature of ruminant brucellosis, the effect of the disease differs by host species. Most infected cows abort once, but shed the bacteria in subsequent parturitions and milk [96]. The rate of abortion in camels is lower than rates in other ruminants—reviewed by Wernery [112], and in experimentally infected camels the symptoms were reported to have been mild and transient [113]. In goats, infections vary from short bouts to persistence [107], and the duration of excretion *Brucella* spp. lasts longer in goats than in sheep [114]. In sheep, the infection pattern is dose dependent and breed related variations have been recorded [107]. The importance of the species as a source of infection to humans could also differ depending on the utility of the host as a milk source and the raw milk consuming tradition of the community. Whilst cow milk is consumed in all systems, camel milk is common in camel rearing pastoral areas. Small ruminants are also used as sources of milk in the pastoral and some crop-livestock system areas [103]. On the whole, given the comparatively higher seroprevalence estimates and number of animals per holder, the impact of animal brucellosis could be substantially higher in the pastoral than the mixed production system, and sizable in the semi-intensive/ intensive systems.

Large scale national level or meta-analytical estimates of brucellosis in sub-Saharan Africa are meager, and comparison of the present estimates with estimates elsewhere is difficult. However, prevalence estimates could differ across countries and time due to factors that may comprise animal, societal, management, and ecological variables [16,115]. A review of brucellosis in SSA is given by Ducrotoy et al. 2015 [116].

### Human brucellosis

**Fig 11:**
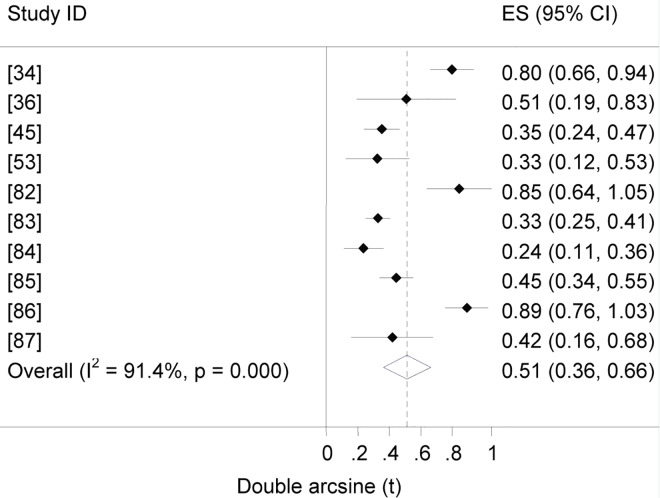
Forest plot of prevalence of brucellosis seropositivity in humans.

Forest plot of the prevalence of brucellosis seropositivity in humans is presented in Fig 11. The overall True Prevalence was estimated at 6.7% (95% CI = 2.4, 12.8). The estimates by subgroup are given in Table 3. The odds ratio was higher in individuals from the pastoral than from the mixed crop-livestock system (OR = 6.7, 95% CI = 3.6, 12.4). The seroprevalence in occupationally exposed individuals (slaughterhouse personnel / butchers /animal health and production workers) was estimated at 1.2% (95% CI = 0, 5.7). The incidence rates in the pastoral and sedentary systems, respectively, were estimated at 160 and 28 per 100 000 person years.

The seroprevalence ratio of brucellosis in humans and animals of the pastoral areas (17.4/4.7) is higher than that of the sedentary system (3.1/1.7). This suggests a higher transmission rate of *Brucella* spp. from animals to humans in the former than in the latter. Similarly, in predominantly pastoral communities of Kenya, a high prevalence of brucellosis seropositivity in humans and livestock, a two to four fold higher prevalence in humans than in animals, and a strong household level association of seropositivity in humans and animals have been described [117]. In rural Ethiopia, livestock delivery is often assisted with bare hands and consuming raw milk is a common practice in a significant segment of the population. Therefore, the seroprevalence estimates in animals and the explicitly low perception of the general population as regards the transmission of zoonotic diseases are evocative of the entrenchment of brucellosis in the pastoral community.

Data on the prevalence of brucellosis seropositivity in individuals occupationally exposed (slaughterhouse personnel / butchers /animal health and production workers) is limited. Among slaughterhouse personnel elsewhere, seroprevalence estimates of 59% (44/75) in Iran [118], 22% (78/360) in Pakistan [119] and as high as 39% (66/170) in Nigeria [120] have been documented. In India, 3.8% (42/1086) in veterinarians and 15% (8/186) in abattoir personnel have been recorded [121]. Differences across countries could be attributed to prevalence differences in animals.

The incidence of human brucellosis in the pastoral areas (160 per 100 000 person years) is considerable. The estimate is higher than rates estimated for several countries [1]: the highest in the Middle East (52–269 per 100 000 person years) and the lowest in Western Europe and the USA (0.02–0.9 per 100 000 person years). In endemic areas asymptomatic brucellosis is common and patients manifest signs when immunocompromised [122]. In a retrospective analysis of data of two health facilities in Uganda (2003–2012), a noticeable prevalence (22%, 381/1732) was recorded in 2012, compared to the years before, and this was associated with an increase in the numbers of individuals tested [123].

Childhood brucellosis in Ethiopia has not been described. In one study on febrile children of sedentary origin, 17(2.6%) were reported to have been seropositive [85]. Although milk could have been the main vehicle of transmission, data on milk contamination levels is lacking. However, levels of 12.6% in informally marketed milk at purchase in Uganda [124], and 18.1% (13/72) in sheep’ and 26.2% (32/122) in goats’ milk in Nigeria [125] have been recorded. In Saudi Arabia, children were reported to have constituted for 21% (115/545) of the total cases of brucellosis, and unpasteurized milk was identified as the main source of infection [126]. In Macedonia, childhood brucellosis (18.8%, 317/1691) was characterized by various organ involvements and a wide spectrum of clinical manifestations [127]. Therefore, given the widespread raw milk consuming habit, it is reasonable to assume that a significant proportion of Ethiopian children, mainly of pastoral origin, could be seroreactors. As most adults are exposed at an early age and do not manifest acute disease, children may account for a higher proportion of patients with acute brucellosis [17].

Human brucellosis in Ethiopia appears to have been under-diagnosed. Thus far there has not been a hospital based retrospective study, and the studies included in this analysis are generally constrained by sample sizes. However, the occurrence of human brucellosis in the rural communities could be linked to the prevalence of brucellosis in animals.

### Need for intervention

The overall data demonstrates the importance of brucellosis and calls for intervention measures -one of which could be livestock vaccination. However, most livestock are kept under an extensive system—where the farming practice is traditional and the road network does not sufficiently go through. In addition, as it currently stands the veterinary sector does not appear to be in a position to carry out a country wide control program. This could be exemplified by the fact that in pastoral areas where brucellosis could have a substantial impact, the animal health care service is for the most part rendered by community based workers. In one instance along the Ethio-Kenya border, the majority of the service providers were reported to have been represented by community based workers (140/167), [128]. Of note are the control programs launched elsewhere in middle and low income countries [97]. For example, in Mongolia livestock vaccination reportedly had brought about a decline in the incidence of human brucellosis in the 1970s; it was modeled to be a cost effective measure at the beginning of the 2000s [129], but in 2010 the rise in the incidence rate of human brucellosis to 229 per 100000 was ascribed to a failure in the functioning of the veterinary sector [130]. In low income countries, *e*x *ante* economic analyses alone may not portray the reality on the ground [2], and several factors may dictate the feasibility and sustainability of a vaccination venture. Therefore, if a countrywide control program is to be a success in Ethiopia, among other factors, the veterinary sector requires a massive investment to strengthen its material and human resources capabilities. Nonetheless, vaccination of livestock in the semi-intensive/ intensive system and breeding farms could be envisaged, albeit the proportion of cattle (< 0.74%) and small ruminants kept under this system is very small [111].

In areas where vaccination could not be put into effect, improvement of the food value chain is a potential intervention measure [2], and public awareness creation and policy measures could help reduce its incidence in both animals and humans. For instance, the diminution in seroprevalence in dairy cattle in Addis Ababa has been credited to the screening and culling practices exercised [52], and implies a potential decrease in the incidence of human brucellosis as well. Similarly, in a stochastic risk assessment of informally marketed milk in Kampala, Uganda, construction of milk boiling centers has been recommended as an intervention measure that could have a substantial impact to shrink the incidence of human brucellosis [124].

### Research needs

This study portrays what has been done hitherto. The pooled estimates could serve the purpose of an input in further studies. Apart from additional seroprevalence studies on human brucellosis, studies on the following would be important to understand the epidemiology of brucellosis and devise control strategies: (i) characterization of *Brucella* spp., (ii) contamination levels of milk with *Brucella* spp., (iii) roles of equines, wildlife and domestic dogs in the epidemiology of brucellosis, (iv) impacts of brucellosis in ruminant productivity and human health, and (v) costs/benefits and feasibility of livestock vaccination in animal production systems context.

### Limitations

As information on the seroprevalence of brucellosis in humans is sparse, the estimates presented here may not reflect the actual occurrence of the disease. Due to lack of uniformity in data presentation, data from all eligible studies have not been made use of. The regression analysis was performed by using data from a subset of studies on animals, and the model does not adequately explain the heterogeneity nor depict the predictive values of the potential explanatory variables.

## Conclusions

The prevalence of brucellosis is higher in goats than in other species. The higher prevalence of brucellosis seropositivity in animals of pastoral origin is suggestive of the importance of human brucellosis in the pastoral systems. The overall data calls for intervention measures that may include public awareness creation, food value chain improvement, and livestock vaccination as appropriate to the systems of animal production. Further studies aimed at generating additional data on issues unaddressed so far, and uniformity in data reporting would be important to further explore and understand the epidemiology of the disease in a National context.

## Supporting Information

S1 Checklist PRISMA checklist. (DOC)

S1 Table Characteristics of included studies. (DOC)

S2 Table Excluded studies. (DOC)
