# Correction: Brucellosis Seropositivity in Animals and Humans in Ethiopia: A Meta-analysis

**DOI:** 10.1371/journal.pntd.0005236

**Published:** 2016-12-20

**Authors:** Getachew Tadesse

Fig 11 is incorrect. The authors have provided a corrected version here.

**Fig 11:**
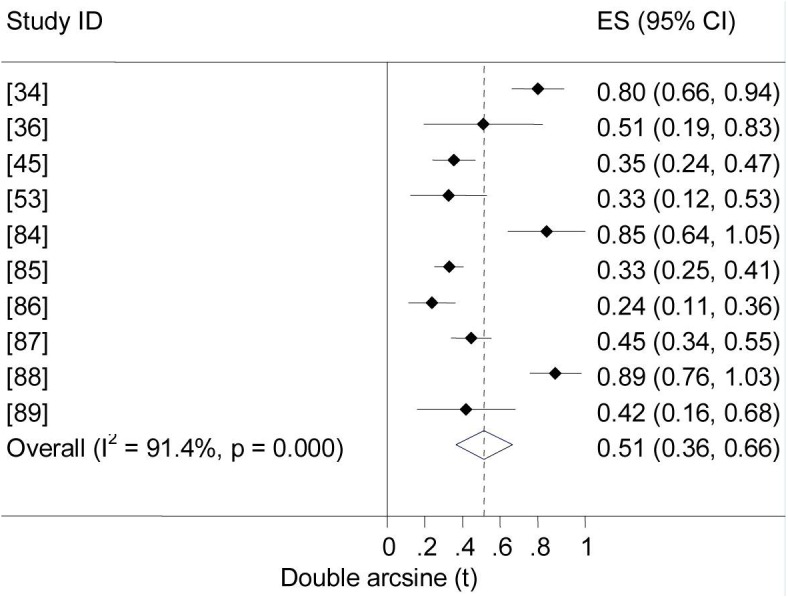
Forest plot of prevalence of brucellosis seropositivity in humans.
