# Supplementary material for: Brucellosis Seropositivity in Animals and Humans in Ethiopia: A Meta-analysis
Source: PLoS Negl Trop Dis. 2016 Oct 28;10(10):e0005006. doi: 10.1371/journal.pntd.0005006 (PMC5085315; doi:10.1371/journal.pntd.0005006)
Supplement: S1 Table — (DOC) [file pntd.0005006.s002.doc]

| Author | Ref. | PY | YS | Region | Host | H/Fn | Ns | P (+) | P (%) |
| --- | --- | --- | --- | --- | --- | --- | --- | --- | --- |
| Balcha & Fentie | [20] | 2011 | 2008-9 | Af,Or,So | Cm | 86 | 1100 | 21 | 1.9 |
| Bekele et al. | [21] | 2013 | 2011-2 | Af | Cm | 100 | 461 | 25 | 5.4 |
| Dele et al. | [22] | 2014 | nr | Or (ex) | Cm,Sp | na | 4244 | 26 | 0.6 |
| Gessese et al.^a^ | [23] | 2014 | 2011-2 | Or (ex) | Cm | na | 1500 | 8 | 0.5 |
| Gumi et al.b | [24] | 2013 | 2008-10 | Or, So | Cm,Ct,Gt | 59,32,34 | 1830 | 64 | 3.5 |
| Habtamu et al.^a,c^ | [25] | 2015 | 2014-5 | Ti | Cm | >120 | 415 | 14 | 3.4 |
| Hadush et al. | [26] | 2013 | nr | Af | Cm | 168 | 1152 | 47 | 4.1 |
| Megersa et al. | [27] | 2005 | 2003-4 | Or | Cm | 250 | 3218 | 58 | 1.8 |
| Megersa et al.^d^ | [28] | 2012 | 2007-8 | Or | Cm,Ct,Gt | 58,107,98 | 2896 | 85 | 2.9 |
| Mohammed et al.^a^ | [29] | 2011 | 2009-10 | Di | Cm | 88 | 573 | 9 | 1.6 |
| Tassew & Kassahun ^a,c^ | [30] | 2014 | 2012-3 | Ti | Cm,Gt | >100 | 450 | 16 | 3.6 |
| Teshome et al. | [31] | 2003 | 2000-1 | Af,Or,So | Cm | nr | 1442 | 60 | 4.2 |
| Tilahun et al. | [32] | 2013 | 2005-6 | So | Cm | 185 | 822 | 20 | 2.4 |
| Warsame et al.^a^ | [33] | 2012 | 2010-1 | Di | Cm | nr | 646 | 10 | 1.5 |
| Zewold & Haileselassie | [34] | 2012 | 2010-1 | Af | Cm | nr | 768 | 58 | 7.6 |
| Adugna et al. | [35] | 2013 | 2007-8 | BG | Ct | 164 | 1152 | 11 | 1.0 |
| Asmare et al. | [36] | 2007 | 2003-4 | Sn | Ct | 347 | 2438 | 47 | 1.9 |
| Asmare et al. | [37] | 2013 | 2011-2 | TAOASD | Ct | 273 | 2334 | 44 | 1.9 |
| Bashitu et al. | [38] | 2015 | 2013-4 | Am,Or | Ct | nr | 415 | 1 | 0.2 |
| Berhe et al. | [39] | 2007 | 2004-5 | Ti | Ct | 26 | 816 | 26 | 3.2 |
| Bsrat et al. | [40] | 2013 | 2006-7 | Or | Ct | 5 | 370 | 7 | 1.9 |
| Degefu et al. | [41] | 2011 | 2008-9 | So | Ct | nr | 435 | 6 | 1.4 |
| Dirar et al. | [42] | 2015 | 2014 | Or | Ct | 105 | 348 | 1 | 0.3 |
| Eshetu et al. | [43] | 2005 | 2002-3 | AA | Ct | 33 | 540 | 54 | 10.0 |
| Haile et al. | [44] | 2010 | 2008-9 | AA | Ct | 295 | 1413 | 67 | 4.7 |
| Hailemelekot et al. | [45] | 2007 | 2004-6 | AA,Or (sl) | Ct | na | 1501 | 17 | 1.1 |
| Hailemelekot et al. | [46] | 2007 | 2004-5 | Am | Ct | 341 | 1944 | 90 | 4.6 |
| Jergefa et al. | [47] | 2009 | 2005-6 | Or | Ct | 176 | 1238 | 37 | 3.0 |
| Kebede et al. | [48] | 2009 | 2004-5 | Or | Ct | 111 | 1136 | 125 | 11.0 |
| Megersa et al.^d^ | [49] | 2011 | 2007-8 | Sn,Or,So | Ct | 134 | 1623 | 51 | 3.1 |
| Mekonnen et al. | [50] | 2010 | 2007-8 | Ti | Ct | 320 | 1968 | 96 | 4.9 |
| Tadele et al. | [51] | 2010 | 2007-8 | Am | Ct | nr | 780 | 4 | 0.5 |
| Tesfaye et al. | [52] | 2011 | 2003-4 | AA | Ct | 51 | 1202 | 18 | 1.5 |
| Tibesso et al. | [53] | 2014 | 2010-1 | Or | Ct | nr | 690 | 30 | 4.3 |
| Tolosa et al.^c^ | [54] | 2010 | 2008-9 | Or | Ct | 4 | 950 | 10 | 1.1 |
| Tolosa et al. | [55] | 2008 | 2003-4 | Or | Ct | 270 | 1305 | 10 | 0.8 |
| Tschopp et al.^b^ | [56] | 2013 | 2011-2 | Or | Ct | 236 | 417 | 7 | 1.7 |
| Tschopp et al.^b,c^ | [57] | 2015 | 2012 | Af, Or | Ct,Gt | 9 | 327 | 67 | 20.5 |
| Yohannes et al. | [58] | 2012 | 2010-1 | Or | Ct | nr | 406 | 8 | 2.0 |
| Adugna et al. | [59] | 2013 | 2011-2 | Af | Sp,Gt | 132 | 1050 | 122 | 11.6 |
| Ali et al. | [60] | 2007 | 2005-6 | Af | Gt | nr | 294 | 54 | 18.4 |
| Ashagrie et al. | [61] | 2011 | 2008-9 | Sn | Gt | 173 | 384 | 16 | 4.2 |
| Ashenafi et al.^c^ | [62] | 2007 | 2005-6 | Af | Sp,Gt | 38 | 1568 | 76 | 4.8 |
| Asmare et al.^a,d^ | [63] | 2013 | 2009-1 | Or, Sn | Gt | 448 | 3315 | 63 | 1.9 |
| Bekele et al.^c^ | [64] | 2011 | 2008-9 | So | Sp,Gt | 64 | 730 | 11 | 1.5 |
| Dabassa et al.^c^ | [65] | 2013 | 2009-10 | Or | Sp,Gt | 89 | 384 | 6 | 1.6 |
| Dedeffo et al.^b^ | [66] | 2015 | 2012 | Or | Sp,Gt | 131 | 840 | 39 | 4.6 |
| Engidaw et al. | [67] | 2015 | 2009-10 | Am | Sp,Gt | nr | 714 | 5 | 0.7 |
| Ferede et al. | [68] | 2011 | 2008-9 | Am | Sp,Gt | nr | 500 | 2 | 0.4 |
| Gebremedhin EZ ^a^ | [69] | 2015 | 2010-2 | Or | Sp | 227 | 1119 | 40 | 3.6 |
| Girmay et al. | [70] | 2013 | 2011-2 | Or (ex) | Sp | na | 2030 | 13 | 0.6 |
| Mohammed et al. | [71] | 2015 | 2013-4 | Or (sl) | Gt | na | 450 | 5 | 1.1 |
| Mohammed et al. | [72] | 2015 | 2011-2 | So | Sp,Gt | nr | 291 | 4 | 1.4 |
| Negash et al.^c,e^ | [73] | 2012 | 2010-1 | Di | Sp,Gt | 49 | 384 | 35 | 9.1 |
| Nigatu et al. | [74] | 2014 | 2011-2 | Or(sl) | Sp,Gt | na | 1000 | 27 | 2.7 |
| Sintayehu et al. | [75] | 2015 | 2004-7 | Af,So,Or,Sn | Sp,Gt | 67 | 6201 | 134 | 2.2 |
| Tadeg et al. | [76] | 2015 | nr | Af | Sp,Gt | 21 | 414 | 57 | 13.8 |
| Teklue et al. | [77] | 2013 | 2011-2 | Ti | Sp,Gt | 53 | 985 | 34 | 3.5 |
| Teshale et al.^b^ | [78] | 2006 | 2004-5 | Af, So | Sp,Gt | nr | 2000 | 193 | 9.7 |
| Tsegay et al. | [79] | 2015 | 2012-3 | Or (sl) | Sp,Gt | na | 853 | 15 | 1.8 |
| Tsehay et al. | [80] | 2014 | 2013-4 | Or, So | Sp,Gt | nr | 420 | 15 | 3.6 |
| Yesuf et al. | [81] | 2010 | 2008-9 | Am | Sp | nr | 800 | 12 | 1.5 |
| Tegegn et al. | [82] | 2016 | 2013-4 | Af | Sp,Gt | 45 | 1190 | 147 | 12.4 |
| Asgedom et al.^b^ | [83] | 2016 | 2013-4 | Or,Sn | Ct | 37 | 804 | 19 | 2.4 |
| Ahmed et al. | [84] | 2008 | 2005-6 | Af | Hu | na | 91 | 15 | 16.5 |
| Animut et al.^b^ | [85] | 2009 | 2006 | Am | Hu | na | 653 | 17 | 2.6 |
| Haileselassie et al.^†^ | [86] | 2011 | 2007-8 | Ti | Hu | na | 246 | 3 | 1.2 |
| Kasshun et al.^b^ | [87] | 2006 | 2002-3 | AA | Hu | na | 336 | 16 | 4.8 |
| Regassa et al.^b^ | [88] | 2009 | nr | Or,Sn,Am | Hu | na | 205 | 38 | 18.5 |
| Tolosa et al. | [89] | 2007 | 2004 | Or | Hu | na | 56 | 2 | 3.6 |
| Zewold & Haileselassie^†^ | [34] | 2012 | 2010-1 | Af | Hu | na | 200 | 30 | 15.0 |
| Asmare et al.^†^ | [36] | 2007 | 2003-4 | Sn | Hu | na | 38 | 2 | 5.3 |
| Hailemelekot et al.^†^ | [45] | 2007 | 2004-6 | Am,Or | Hu | na | 305 | 9 | 3 |
| Tibesso et al.^†^ | [53] | 2014 | 2010-1 | Or | Hu | na | 93 | 2 | 2.2 |
| AA, Addis Ababa; Af, Afar; Am, Amhara; BG, BeneShangul-Gumiz; Ct, cattle; Di, Dire Dawa; Gt, Goats; H/Fn, herd/flock number; ex, export animals; Hu, human; na, not applicable; nr, not reported; Ns, number of samples; Or, Oromia; Py, Publication year; Ref, Reference; S, sedentary; sl, slaughtered animals; Sn, Southern Nations and Nationalities; Sp, Sheep; So, Somali; TAOASD, (Tigray, Amhara, Oromia, Addis Ababa, Southern Nations and Nationalities, Dire Dawa); Ti, Tigray; Ys, Year of study.  ^†^Studies that reported brucellosis on animals and humans.  ^a^Data included depending on proximity to main pastoral areas: Bale in Borana [23]; Mehoni in Afar [25,30]; Diredawa in Somali [29,33,73]; Fentale in Afar [63,69].  ^b^Tests other than RBT-CFT in series.  ^c^Herd/flock number is based on villages [54,57,62,64] and owners interviewed [25, 30, 65,73]  ^d^The numbers of seropositive animals by category were calculated [28,49, 63].  ^e^The data is grouped in the pastoral system. | | | | | | | | | |
