# Supplementary material for: Brucellosis Seropositivity in Animals and Humans in Ethiopia: A Meta-analysis
Source: PLoS Negl Trop Dis. 2016 Oct 28;10(10):e0005006. doi: 10.1371/journal.pntd.0005006 (PMC5085315; doi:10.1371/journal.pntd.0005006)
Supplement: S2 Table — (DOC) [file pntd.0005006.s003.doc]

| No. | PR | Author | Species | Reference |
| --- | --- | --- | --- | --- |
| 1 | RW | Addis M. | Overall | Public Policy and Administration Research.2015; 5: 68-83 |
| 2 | RW | Yohannes et al. | Overall | Afr J Microbiol Res.2013; 1150-1157. |
| 3 | RW | Asmare et al. | Cattle | Trop Anim Health Prod. 2014; 46:1341-1350. |
| 4 | BT | Domenech et al. | Camel | Rev Elev Med vét Pays trop. 1977; 30: 141.142. |
| 5 | BT | Richard L. | Camel | Trop Anim Health Prod. 1980; 35:381-389. |
| 6 | BT | Domenech & Lefevere | Cattle | Rev Elev Med vét Pays trop.1974; 27: 385-395. |
| 7 | BT | Asfaw et al. | Cattle | Bull Anim Health Prod Afr. 1998:46: 217-224. |
| 8 | BT | Bekele et al. | Cattle | Bull Anim Health Prod Afr. 1998; 48: 13-17. |
| 9 | BT | Bekele et al. | Cattle | Bull Anim Health Prod Afr. 1997: 37, 97-98. |
| 10 | BT | Bekele et al. | Sp, Gt | Bull Anim Health Prod Afr. 1998; 38: 23-25. |
| 11 | BT | Alemayehu E | Human | Ethiop Med J. 1998; 19:21-24. |
| 12 | BT | Seboxa T. | Human | Ethiop Med J. 1982; 20:189-192. |
| 13 | BT | Cramlet & Berhanu | Horse | [Vet Med Small Anim Clin.](http://www.ncbi.nlm.nih.gov/pubmed/107647) 1979; 74:195-199. |
| 14 | DR [31] | Teshome & Molla | Camel | J Camel Pract Res. 2002; 9:125-128. |
| 15 | DR [23] | Abebe et al. | Camel | Folia Veterinaria. 2014; 58: 5-8. |
| 16 | DR [36] | Asmare et al. | Cattle | Afr J Agric Res. 2010; 5: 257-263. |
| 17 | DR [37] | Asmare et al. | Cattle | Trop Anim Health Prod. 2014; 46:961-966. |
| 18 | DR [37] | Asmare et al. | Cattle | Epidemiol Infect. 2013; 141:1772-1780. |
| 19 | DRp [50] | Hailesellasie et al. | Cattle | Vet Med Int. 2011; 354943. doi:10.4061/2011/354943. |
| 20 | DRp [46] | Hailemelekot et al. | Cattle | Ethiop Vet J. 2007; 11: 85-100. |
| 21 | DR [28] | Megersa et al. | Cm,Ct,Gt | Trop Anim Health Prod. 2011; 43:651–656 |
| 22 | DR [67] | Engidaw et al. | Sp, Gt | WJPLS. 2015; 1: 12-23. www.wjpls.org |
| 23 | SF | Berhe & Gangwar | Cattle | IJSN. 2011: 2: 692- 697. [www.science](http://www.science/)andnature.org. |
| 24 | RB | Abebe et al. | Cattle | Zoonoses Public Health. 2010; 57: 367–374. |
| 25 | RB | Amenu et al. | Ct, Sp,Gt | Tropicultura. 2010; 28: 205-210. |
| 26 | RB | Benti & Zewdie | Cattle | J Adv Vet Anim Res. 1:182-188. |
| 27 | RB | Dinka & Chala | Cattle | American-Eurasian J Agric & Environ Sci. 2009; 6:508-512. |
| 28 | RB | Dinka H. | Cattle | J Vet Med Anim Health. 2013; 5:113-117. |
| 29 | RB | Molla & Delil | Ct, Sp,Gt | Trop Anim Health Prod. 2015; 47:45-51. |
| 30 | RB | Lemu et al. | Sp, Gt | Ethiopian Int J Multidiscip Res. 2014; 1: 14-18. |
| 31 | RB | Yilma M. | Cattle | Immunome Res 2016; 12: 105. doi:10.4172/1745-7580.10000105 |
| 32 | RB | Kebeta et al. | Pigs | J Veterinar Sci Technolo. 2015; 6:2. doi.org/10.4172/2157-7579.1000215. |
| 33 | NF | Ibrahim et al. | Cattle | Trop Anim Health Prod.2010; 42:35-40. |
| 34 | NF | Degefa et al. | Cattle | Global Veterinaria. 2011; 7: 45-53. |
| 35 | NF | Alemu et al | Cattle | Acad J Anim Diseases. 2014; 3: 27-32. |
| 36 | UN | Tesfaye et al. | Sp,Gt | Libyan Agric Res Cent J Int. 2012; 3: 47-52. |
| 37 | UN | Bedane et al. | Cattle | Libyan Agric Res Cent J Int. 2012; 3: 53-59. |
| 38 | NF | Yosef & Nardos | Sheep | Indian J Comp Microbiol Immunol Infect Dis.2010; 31:44-47. |
| 39 | NF | Geresu et al. | Cattle | Afr J Microbiol Res. 2016; 10: 203-213. |
| 40 | RW | Yilma et al. | Overall | Achievem Life Sci 2016; doi.org/10.1016/j.als.2016.05.008 |
| BT, before 2000; Cm, camel; Ct, cattle; DR, data in reference [ ]; DRp, part of data in reference [ ]; Gt, goat; NF, data not figured out; PR, primary reason; RB, RBPT test alone; RW, review; UN, unavailable (URL /larcji/larcji.htm was not found); Sp, sheep. | | | | |
